# Evolution in the management of acute cholecystitis in the elderly: population-based cohort study

**DOI:** 10.1007/s00464-018-6092-5

**Published:** 2018-07-25

**Authors:** Tom Wiggins, Sheraz R. Markar, Hugh Mackenzie, Sara Jamel, Alan Askari, Omar Faiz, Stavros Karamanakos, George B. Hanna

**Affiliations:** 1 Department Surgery & Cancer, Imperial College London, London, UK; 2 St Mark’s Hospital and Academic Institute, Harrow, UK; 3 Basildon University Hospitals NHS Trust, Basildon, UK; 4 Division of Surgery, Department of Surgery and Cancer, St Mary’s Hospital, Imperial College London, 10th Floor QEQM Building, South Wharf Road, London, W2 1NY UK

**Keywords:** Cholecystitis (MeSH), Cholecystitis, Acute (MeSH), Aged (MeSH)

## Abstract

**Background:**

Acute cholecystitis is a life-threatening emergency in elderly patients. This population-based cohort study aimed to evaluate the commonly used management strategies for elderly patients with acute cholecystitis as well as resulting mortality and re-admission rates.

**Methods:**

Data from all consecutive elderly patients (≥ 80 years) admitted with acute cholecystitis in England from 1997 to 2012 were captured from the Hospital Episode Statistics database. Influence of management strategies upon mortality was analyzed with adjustment for patient demographics and treatment year.

**Results:**

47,500 elderly patients were admitted as an emergency with acute cholecystitis. On the index emergency admission the majority of patients (*n* = 42,620, 89.7%) received conservative treatment, 3539 (7.5%) had cholecystectomy, and 1341 (2.8%) underwent cholecystostomy. In the short term, 30-day mortality was increased in the emergency cholecystectomy group (11.6%) compared to those managed conservatively (9.9%) (*p* < 0.001). This was offset by the long-term benefits of cholecystectomy with a reduced 1-year mortality [20.8 vs. 27.1% for those managed conservatively (*p* < 0.001)]. Management with percutaneous cholecystostomy had increased 30-day and 1-year mortality (13.4 and 35.0%, respectively). The annual proportion of cholecystectomies performed laparoscopically increased from 27% in 2006 to 59% in 2012. Within the cholecystectomy group, laparoscopic approach was an independent predictor of reduced 30-day mortality (OR 0.16, 95% CI 0.10–0.25). Following conservative management, there were 16,088 admissions with further cholecystitis. Only 11% of patients initially managed conservatively or with cholecystostomy received subsequent cholecystectomy.

**Conclusion:**

Acute cholecystitis is associated with significant mortality in elderly patients. Potential benefits of emergency cholecystectomy in selected elderly patients include reduced rate of readmissions and 1-year mortality. Laparoscopic approach for emergency cholecystectomy was associated with an 84% relative risk reduction in 30-day mortality compared to open surgery.

**Electronic supplementary material:**

The online version of this article (10.1007/s00464-018-6092-5) contains supplementary material, which is available to authorized users.

The United Kingdom has an increasing elderly population and the proportion of people over the age of 80 is continuing to grow [1]. This patient population represents a clinical challenge and places increasing demands upon healthcare resources. Gallstones are one such disease which becomes more prevalent with age, affecting up to 30% of individuals over 60 years [2, 3], and 80% of institutionalized individuals over age 90 [4]. Acute cholecystitis is one of the most common complications of gallbladder stones and often necessitates emergency hospital admission for treatment.

Cholecystectomy represents the only definitive cure for symptomatic gallstone-related disease. In general, emergency cholecystectomy for acute cholecystitis is associated with reduced long-term biliary complications, shorter total admission length of stay, and lower overall treatment costs [5–11]. Clinical guidelines in the United Kingdom reflect this with the National Institute of Clinical Excellence recommending patients with acute cholecystitis receive cholecystectomy within 1 week of diagnosis [12]. However, the management of elderly patients with acute cholecystitis represents a complex challenge due to the balance of benefits from emergency cholecystectomy versus the increased potential risk of perioperative morbidity and mortality [13]. The greater burden of comorbid disease in elderly patients leads to reduced physiological reserve and increased susceptibility to perioperative complications such as myocardial injury and respiratory compromise [14]. Increasing age has previously been identified as a factor which significantly reduces the likelihood of emergency cholecystectomy being undertaken [15, 16]. Despite these compromising factors, institutional studies (with small sample sizes) have demonstrated that emergency cholecystectomy for acute cholecystitis in selected elderly patients can be performed safely [17], and is associated with improved 2-year survival and reduced overall healthcare costs [18]. In one study, 38% of elderly patients undergoing conservative management for cholecystitis were readmitted with gallstone-related disease compared to only 4% readmission rate in those receiving cholecystectomy on index admission [18]. Although management of cholecystitis in patients over the age of 65 has been investigated previously [18–21], treatment of those specifically at the extremes of age (over 80 years old) remains largely unstudied.

The objective of this national population-based cohort study was to establish the management strategies employed and clinical outcomes for patients over the age of 80 years presenting with acute cholecystitis in England.

## Materials and methods

Data were derived from the Hospital Episode Statistics (HES) database [22]. This is an administrative dataset that collects patient-level data from all National Health Service (NHS) hospitals in England. It captures all patients treated in public sector hospitals and a minority of patients treated in privately funded institutions. Patients are linked to a unique HES identifier, which allows all of their hospital admissions to be tracked throughout the dataset. Permissions for the comparison of anonymized administrative data were obtained from the National Information Governance Board for Health and Social Care in England.

### Coding of data

Relevant International Classification of Disease (ICD-10) codes were used to identify all patients over the age of 80 years who were admitted as an emergency for the treatment of acute cholecystitis (Supplementary Material), between 1st January 1997 and 31st December 2012. Patients primarily admitted with other gallstone-related disorders, including biliary colic, acute cholangitis, and acute pancreatitis were excluded from this study. Only patients identified as emergency admissions were included in this analysis using the admission codes (method of admission 21–28). Readmissions following index emergency admission with further gallstone-related disease were identified using relevant ICD-10 codes (Supplementary Material). Local verification of ICD-10 codes used for diagnosis and patient allocation was performed as part of the quality assurance for the data included.

### Allocation to treatment groups

Treatments were identified using the Office of Population Censuses and Surveys Classification of Surgical Operations and Procedures 4th revision (OPCS-4) codes (Supplementary Material). Treatment groups included the following:

1. Conservative management (defined as best supportive medical treatment alone in the absence of any formal surgical, endoscopic, or radiological intervention).
2. Cholecystectomy on index admission.
3. Percutaneous cholecystostomy placement on index admission.

All patients requiring initial intervention with endoscopic retrograde cholangio-pancreatogram (ERCP) were excluded from analysis due to the potential influence of common bile duct stones or the presence of ascending cholangitis upon outcomes.

Within the cholecystectomy group, patients were subdivided into those receiving laparoscopic cholecystectomy and those undergoing open surgery. Patients who had initial attempt at a laparoscopic approach followed by conversion to open were included in the laparoscopic surgery group (based on an intention to treat analysis). Post-operative complications were defined as bile duct injury (defined by need for biliary reconstruction) or need for post-operative ERCP (as treatment of post-operative bile leak or retained stones). These interventions were identified through the relevant OPCS-4 codes (Supplementary Material).

### Exposure

The exposure under investigation was index admission management strategy: either conservative management, cholecystectomy, or percutaneous cholecystostomy in patients over the age of 80 with acute cholecystitis.

### Outcomes

Primary outcome measures were 30-, 90-day, and 1-year mortality. Mortality was identified by linking HES data with data from the Office for National Statistics (ONS). The process of data linkage was performed centrally using the unique patient NHS number, which permits linkage of data between patient datasets. Secondary measures included patient readmission with gallstone-related disease following conservative management or percutaneous cholecystostomy tube placement, and complications within the cholecystectomy group (need for re-admission, bile duct injury, need for post-operative ERCP).

### Statistical analysis

Statistical analysis was performed using SPSS version 23.0 software (Statistical Package for the Social Sciences software, Version 23, SPSS Chicago (IL), USA). Univariate comparisons between treatment groups were made with Chi-squared test for discrete variables and Kruskal–Wallis test for continuous variables. Multivariable logistic regression analyses were performed to evaluate the positive or negative association of emergency cholecystectomy with 30-, 90-day, and 1-year mortality. Confounding factors adjusted for in this analysis included the following: sex (male or female), Charlson Comorbidity Index score (< 2 or ≥ 2), and time period of primary index admission (either 1997–2004 or 2005–2012).

## Results

### Population characteristics

Over the 16-year period from 1997 to 2012, there were 47,500 patients over the age of 80 admitted with acute cholecystitis in England. Numbers of admissions increased over time with an average 2383 admissions per year between 1997 and 2004, increasing to an average 3555 emergency admissions per year between 2005 and 2012. Median age was 85 (range 80–100). Nearly, two-thirds of patients were male (63 vs. 37% females). Eighty-three percent of patients had a Charlson Comorbidity Index below 2. Table 1 shows the patient number and demographics for each management strategy evaluated (conservative management, emergency cholecystectomy or percutaneous cholecystostomy).

**Table 1:** Results summary: Overall results including patient demographics, 30-, 90-day, and 1-year mortality for each treatment group

	Overall	Conservative (%)	Cholecystectomy (%)	Cholecystostomy (%)	*p* Value
Patient number	47,500	42,620 (89.7)	3539 (7.5)	1341 (2.8)	
Age [median (range)]	85 (80–100)	85 (80–100)	83 (80–100)	85 (80–100)	< 0.001
Gender					0.640
Male	29,952 (63.1)	26,905 (63.1)	2210 (62.4)	837 (62.4)	
Female	17,548 (36.9)	15,715 (36.9)	1329 (37.6)	504 (37.6)	
Charlson					< 0.001
< 2	39,621 (83.4)	35,408 (83.1)	3097 (87.5)	1116 (83.2)	
≥ 2	7879 (16.6)	7212 (16.9)	442 (12.5)	225 (16.8)	
Year					< 0.001
1997–2004	19,062 (40.1)	16,824 (39.5)	1789 (50.6)	449 (33.5)	
2005–2012	28,438 (59.9)	25,796 (60.5)	1750 (49.4)	892 (66.5)	
30-day mortality	4829 (10.2)	4240 (9.9)	409 (11.6)	180 (13.4)	< 0.001
90-day mortality	7730 (16.3)	6876 (16.1)	552 (15.6)	302 (22.5)	< 0.001
1-year mortality	12,768 (26.9)	11,563 (27.1)	736 (20.8)	469 (35.0)	< 0.001

### Management strategy

The majority of elderly patients presenting with acute cholecystitis were treated conservatively (89.7%, *n* = 42,620). Only 7.5% (*n* = 3539) of patients over the age of 80 received emergency cholecystectomy on their index admission with acute cholecystitis. Over the study period 1341 (2.8%) elderly patients with acute cholecystitis had percutaneous cholecystostomy performed.

**Fig. 1:**
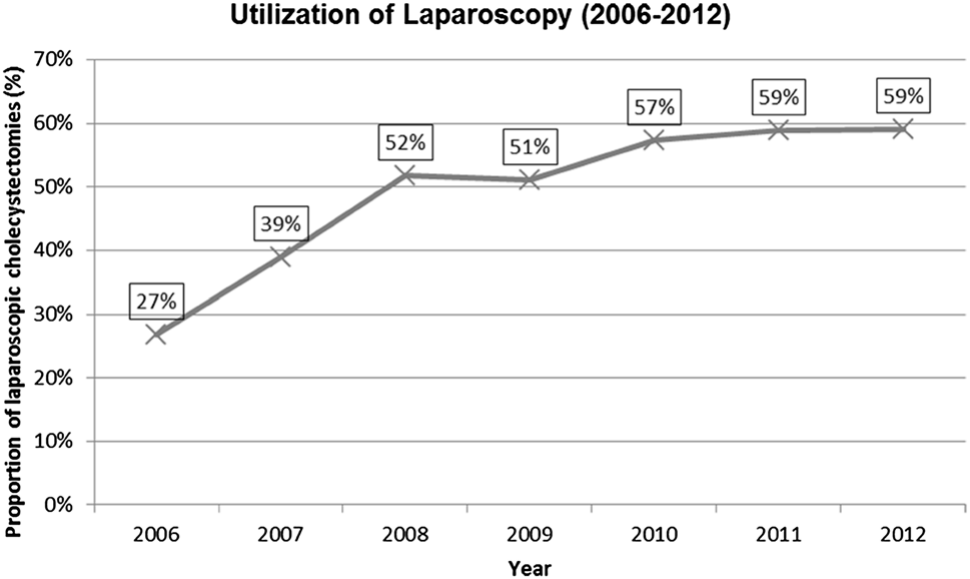
Increasing utilization of laparoscopic approach for cholecystectomy from 2006 to 2012

The proportion of patients treated conservatively was similar between the study periods (1997–2004: 88%; 2005–2012: 91%), whereas the proportion of cases receiving emergency cholecystectomy appeared to reduce over time (1997–2004: 9.4%; 2005–2012: 6.2%). Rate of laparoscopic approach for cholecystectomy increased substantially over time. There were no recorded emergency laparoscopic cholecystectomies in this age group prior to 2006. In subsequent years, the rate of laparoscopic approach increased from 27% in 2006 to 59% in 2012 (see Fig. 1). Utilization of percutaneous cholecystostomy increased slightly over time with a usage in 2.4% of cases between 1997 and 2004 compared to 3.1% from 2005 to 2012.

Median age in the conservative management group was 85 (range 80–100). Median age was the same in the percutaneous cholecystostomy group [median age 85 (range 80–100)], whereas patients undergoing cholecystectomy on index admission were significantly younger [83 (range 80–100 (*p* < 0.001))]. Patients treated with emergency cholecystectomy had significantly fewer co-morbidities (Charlson Comorbidity Index below 2 in 87.5% of the emergency cholecystectomy group versus 83.1% in the conservative group, and 83.2% in the percutaneous cholecystostomy group) (*p* < 0.001) (Table 1).

### Mortality

**Fig. 2:**
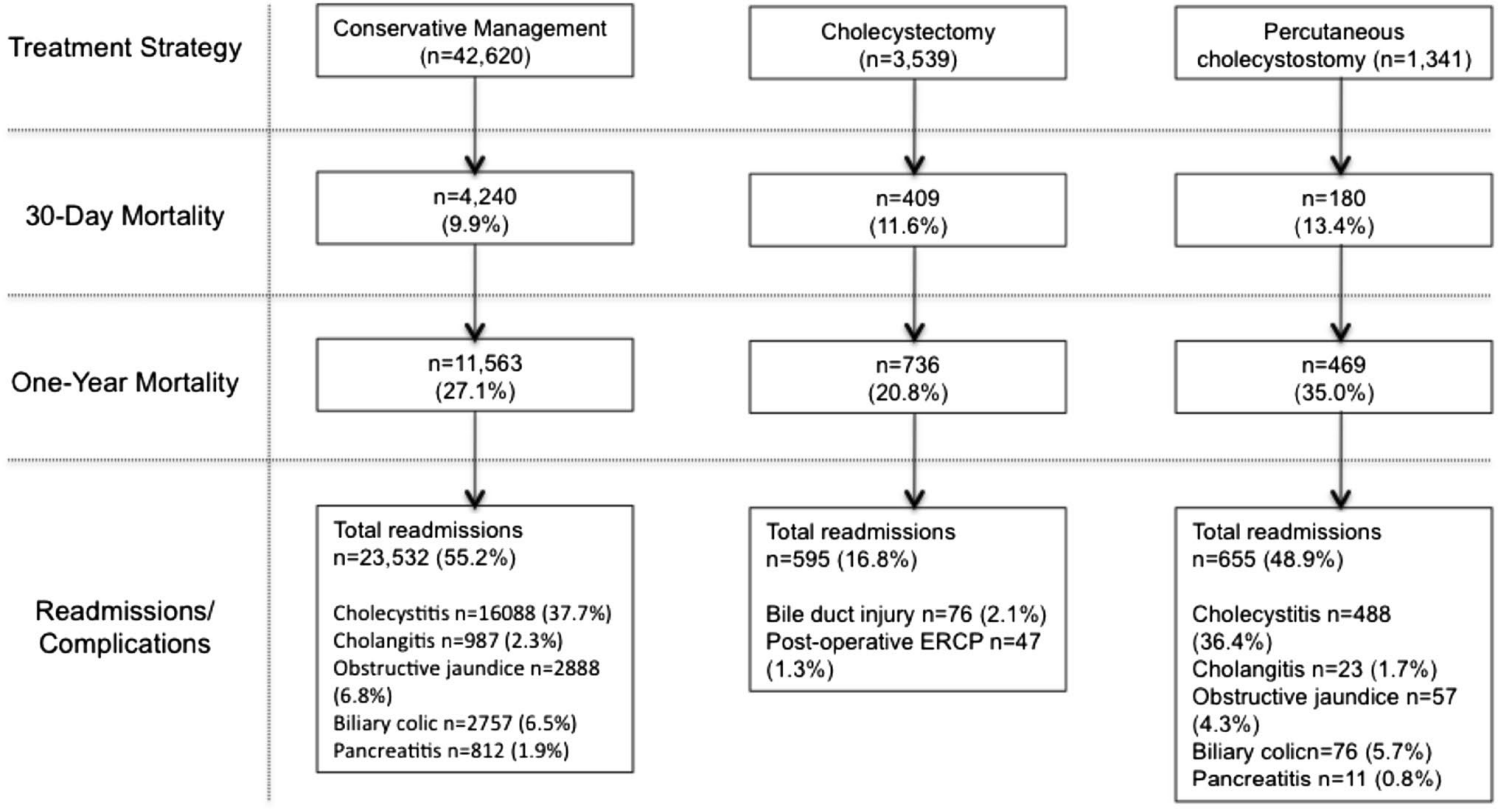
Flow-chart detailing 30-day mortality, 1-year mortality, and readmissions/complications for each treatment strategy

Overall 30-day mortality in the conservative management group was 9.9% (*n* = 4240). In comparison, patients treated with emergency cholecystectomy had a greater 30-day mortality rate [11.6% (*n* = 409)], but the group with the greatest mortality risk was the percutaneous cholecystostomy cohort [13.4% (*n* = 180) (*p* < 0.001)]. When investigating 90-day mortality rates, the cholecystectomy group had a similar outcome to those managed conservatively (15.6% in cholecystectomy group versus 16.1% in the conservative group). The highest mortality rate at 90 days remained in the patients receiving treatment with percutaneous cholecystostomy (22.5%) (*p* < 0.001). Over longer term follow-up, the cholecystectomy group had the lowest mortality rate at 1-year [20.8% in cholecystectomy group; 27.1% in conservative group; 35% in percutaneous cholecystectomy group (*p* < 0.001)] (Table 1; Fig. 2).

### Readmissions

In patients managed conservatively (*n* = 42,620), there were 16,088 readmissions (37.7%) with acute cholecystitis over the study period. Conservatively managed patients had an additional 2757 readmissions with biliary colic, 987 readmissions with cholangitis, 2888 incidences of obstructive jaundice, and 812 episodes of pancreatitis. In total during the study period, there were 23,532 readmissions with gallstone-related disease following conservative management of cholecystitis. This represents 55.2% of total cases (Fig. 2). Of patients managed conservatively only 11.2% (*n* = 4762) went on to receive elective delayed cholecystectomy.

Following cholecystectomy, 16.8% of cases (*n* = 595) required emergency readmission. Forty-seven cases (1.3%) required post-operative ERCP for retained stones or bile leak. Seventy-six patients (2.1%) required common bile duct reconstruction following intra-operative injury (Fig. 2). Patients who had a bile duct injury and reconstruction did not have a significant increase in mortality compared to those patients within the cholecystectomy group who did not have a bile duct injury [30-day mortality 12.8 vs. 11.4%, respectively (*p* = 0.700); 90-day mortality: 14.1 vs. 15.5%; (*p* = 0.728); 1-year mortality 26.9 vs. 20.7%; *p* = 0.177].

Following percutaneous cholecystostomy no patients required further intervention with additional cholecystostomy tube placement or ERCP. However, there were 655 readmissions with gallstone-related disease (cholecystitis *n* = 488; cholangitis *n* = 23; obstructive jaundice *n* = 57; biliary colic *n* = 76; pancreatitis *n* = 11) (Fig. 2). Of patients receiving percutaneous cholecystostomy, only 11.7% went on to receive interval cholecystectomy.

### Multivariate analysis

In multivariate analysis increasing age, Charlson Comorbidity Index above 2 and treatment in the second time period (2005–2012) were associated with increased 30-, 90-day, and 1-year mortality. Male gender was associated with increased mortality at 30 and 90 days. Treatment with cholecystectomy or percutaneous cholecystostomy was also associated with increased mortality at 30 and 90 days. However, 1-year mortality was reduced in those patients undergoing cholecystectomy relative to those treated conservatively (OR 0.85; 95% CI 0.78–0.93; *p* < 0.001). Percutaneous cholecystostomy remained associated with increased mortality at 1-year (OR 1.46; 95% CI 1.29–1.64; *p* < 0.001) (Table 2).

**Table 2:** Multivariate analysis of all patients: Multivariate analysis of all cholecystitis patients including effect of treatment modality upon 30-, 90-day, and 1-year mortality

Variable	30-Day mortality			90-Day mortality			1-Year mortality		
	Odds ratio	95% Confidence interval	*p* Value	Odds ratio	95% Confidence interval	*p* Value	Odds ratio	95% confidence Interval	*p* Value
Age (continuous)	1.09	1.08–1.10	< 0.001	1.09	1.09–1.10	< 0.001	1.10	1.10–1.11	< 0.001
Sex									
Male (Ref.)	1	–	0.012	1	–		1	–	
Female	0.92	0.86–0.98		0.91	0.87–0.96	0.001	0.99	0.95–1.04	0.646
Charlson									
< 2 (Ref.)	1	–	< 0.001	1	–		1	–	
≥ 2	2.84	2.66–3.04		2.89	2.73–3.06	< 0.001	2.84	2.69–2.99	< 0.001
Year									
1997–2004 (Ref.)	1	–	0.010	1	–		1	–	
2005–2012	1.09	1.02–1.16		1.08	1.02–1.13	0.007	1.05	1.01–1.10	0.023
Treatment									
Conservative (Ref.)	1	–		1	–		1	–	
Cholecystectomy	1.47	1.31–1.64	< 0.001	1.18	1.07–1.30	< 0.001	0.85	0.78–0.93	< 0.001
Cholecystostomy	1.41	1.19–1.66	< 0.001	1.53	1.33–1.75	< 0.001	1.46	1.29–1.64	< 0.001

In a subset analysis of patients who underwent emergency cholecystectomy, increasing age, Charlson Comorbidity Index above 2 and treatment between 2005 and 2012 were again associated with increased mortality. Utilization of a laparoscopic approach for cholecystectomy was associated with substantially improved outcomes with an 84% relative risk reduction in 30-day mortality compared to those treated with open cholecystectomy (Table 3).

**Table 3:** Multivariate analysis of cholecystectomy patients: 30-, 90-day, and 1-year mortality for cholecystectomy patients

Variable	30-Day mortality			90-Day mortality			1-year mortality		
	Odds ratio	95% Confidence interval	*p* Value	Odds ratio	95% Confidence interval	*p* Value	Odds ratio	95% Confidence interval	*p* Value
Age (continuous)	1.10	1.07–1.13	< 0.001	1.11	1.08–1.14	< 0.001	1.10	1.08–1.13	< 0.001
Sex									
Male (Ref.)	1	–		1	–		1	–	
Female	1.19	0.96–1.49	0.118	1.08	0.89–1.32	0.436	1.18	0.99–1.41	0.064
Charlson									
< 2 (Ref.)	1	–		1	–		1	–	
≥ 2	3.19	2.47–4.11	< 0.001	3.61	2.86–4.56	< 0.001	3.68	2.96–4.57	< 0.001
Year									
1997–2004 (Ref.)	1	–		1	–		1	–	
2005–2012	1.45	1.16–1.81	0.001	1.36	1.11–1.67	0.003	1.24	1.03–1.50	0.020

## Discussion

This study of 47,500 patients over the age of 80 admitted as an emergency with acute cholecystitis has demonstrated an increasing admission rate over time, with the majority of patients (89.7%) treated conservatively on their index admission. However, this population-based study has revealed poor clinical outcomes in elderly patients for a common benign condition which is associated with very good outcomes in the general population [23, 24]. Overall 30-day mortality was 10%, increasing to 16% at 90-day and 27% after 1 year. Of those patients managed conservatively over half required readmission with further gallstone-related complications. Major technical complications of cholecystectomy included bile duct injury requiring reconstruction in 2.1% of patients and biliary leakage or retained stones necessitating ERCP in 1.3%.

Increasing age significantly reduces the likelihood of emergency cholecystectomy being undertaken [15, 16, 21]. Age of 80 years or older has been demonstrated to be independently associated with increased morbidity following cholecystectomy for acute cholecystitis (31 vs. 13%; *p* = 0.01) as well as greater risk of conversion from a laparoscopic to open procedure (21 vs. 7%; *p* = 0.001) [25]. However, it has also been demonstrated that undertaking cholecystectomy for elderly patients during initial hospital admission will prevent further episodes of gallstone-related disease, reduce readmission rates, and is associated with lower overall healthcare costs [18].

A recent observational study has identified that in the United Kingdom a smaller proportion of patients with acute cholecystitis (of any age group) receive emergency cholecystectomy on their index admission compared to the United States (Rate of emergency cholecystectomy in UK 15.7 vs. 52.7% in USA) [26]. Previous studies from the United Kingdom have recommended that elderly patients with acute cholecystitis should be managed conservatively due to the potentially significant risk of post-operative morbidity in these patients, and interval cholecystectomy be reserved for those with recurrent episodes of cholecystitis [19]. In the current study, those patients managed conservatively did have significantly reduced 30- and 90-day mortality compared to those receiving emergency cholecystectomy. The 30- and 90-day mortality rates following emergency cholecystectomy in these elderly patients were 11.6 and 15.6%, respectively, which is greater than that reported in a recent pooled analysis of outcomes in patients over 75 undergoing emergency cholecystectomy (pooled mortality rate of 3.5%) [27]. The cause of this difference in mortality rate is unclear but it is important to consider that the current study included 3539 elderly patients undergoing emergency cholecystectomy, whereas this pooled analysis only included data from 592 patients [27]. Despite the 30-day mortality rate of 11.6% for elderly patients undergoing cholecystectomy in the current study, the utilization of a laparoscopic approach for surgery was associated with an 84% relative risk reduction in 30-day mortality compared to open surgery. This is consistent with previous findings [28], and probably largely driven by reduced rates of post-operative pneumonia associated with laparoscopic surgery due to improved pulmonary function post-operatively [29]. At 1-year, patients treated with cholecystectomy had reduced mortality relative to those treated conservatively (20.8 vs. 27.1%). This is most likely to be a result of the prevention of further gallstone-related complications following cholecystectomy. Fifty-five percent of patients managed conservatively required readmission due to further gallstone-related complications which is similar to previously reported readmission rates following conservative treatment in elderly patients [18]. The benefits of surgery at one year must be weighed against the increased short-term mortality as demonstrated in the current study.

The cohort of patients receiving percutaneous cholecystostomy may have the most severe form of cholecystitis and have been considered unsuitable for any surgical intervention. The Tokyo guidelines have recently been updated to recommend the potential consideration of early laparoscopic cholecystectomy in patients with severe cholecystitis (Severity grade III) if appropriate experience is available and patients do not have any negative predictive factors such as jaundice, neurological dysfunction of respiratory failure [30]. However, the utilization of percutaneous cholecystostomy is likely to have been more common in these patients with severe cholecystitis and evidence of organ dysfunction. This factor would potentially explain the increased 30- and 90-day mortality identified in these patients. The use of percutaneous cholecystostomy has previously been suggested as a bridging procedure to resolve sepsis in high-risk patients prior to early cholecystectomy [31, 32]. However, in the current study, only 11.7% of patients receiving percutaneous cholecystostomy went on to undergo cholecystectomy. Previous studies have shown that 46% of patients undergoing percutaneous cholecystostomy alone (without subsequent cholecystectomy) will develop further episodes of cholecystitis within 3 years [33].

Despite the inherent limitations of a national administrative database study, the population-based design with virtually complete inclusion of all eligible patients over the age of 80 in England is a significant strength of this study. The large sample size in a population of patients at the extremes of age allows the assessment of overall outcomes for these patients at a national level. The complete-follow-up of all patients and adjustment for several relevant confounding factors are further advantages. Due to the nature of this national database and retrospective design of the study, it was not possible to adjust for patient physiological status at the time of admission, or clinical severity of acute cholecystitis. It was also not possible to identify specific forms of cholecystitis that are associated with higher rates of complications such as gangrenous or emphysematous cholecystitis. As a large national database study, the results generated are dependent upon the reliability of the methodology and accuracy of data collection, which is a limitation shared by all national administrative datasets. Additionally, the study period of 16 years is relatively long and there may have been changes in practice over this period in unmeasured factors (such as availability of intensive care facilities or access to radiology services), which may have affected patient outcomes. However, to account for the influence of presentation in a later time period, this has been incorporated as a confounding variable within the regression model presented in Tables 2 and 3. This study identified that 2.1% of elderly patients undergoing emergency cholecystectomy suffered a bile duct injury which required reconstruction. Unfortunately due to the nature of the database utilized, it was not possible to classify the severity of the bile duct injury according to the Strasberg classification [34], and the precise method of reconstruction was not directly available for analysis.

Cholecystectomy for acute cholecystitis is generally perceived as being associated with low peri-operative mortality risk (below 1% for the general population [24]). However, within a high-risk patient group such as the elderly, mortality significantly increased with a 30-day mortality of 11.6% in the current study. This compares unfavorably to procedures which are generally deemed ‘high-risk’ such as esophagectomy despite the fact these operations are undertaken on elderly patients with lower mortality rates than those associated with emergency cholecystectomy in the current analysis [35, 36]. Outcomes from major elective procedures have been improved substantially in recent years in part as a result of the adoption of Enhanced Recovery After Surgery (ERAS) programs [37, 38]. A cultural-shift to view cholecystectomy (either laparoscopic or open) in elderly patients as a major procedure requiring similar peri- and post-operative care as more substantial procedures such as esophagectomy or major colorectal resections may lead reduced mortality rates. This would include adopting the principles of ERAS that have been demonstrated to significantly improve outcomes following major abdominal surgery [37, 38]. It may be possible to improve outcomes for these patients via the development of a standardized clinical pathway which incorporates the principles of ERAS such as pre-operative carbohydrate loading, avoidance of fluid overloading intra-operatively, resumption of oral feeding post-operatively, early mobilization and intensive chest physiotherapy [39–41]. There should also be a low-threshold for patients to be managed in a high-dependency unit following surgery. By adopting this form of approach, it is anticipated that similar improvements in post-operative outcomes as those seen in other procedures such as esophagectomy and major colorectal resection could be translated to these ‘high-risk’ patients undergoing cholecystectomy.

## Electronic supplementary material

Below is the link to the electronic supplementary material.

Supplementary material 1 (DOCX 14 KB)
