# Supplementary material for: Evolution in the management of acute cholecystitis in the elderly: population-based cohort study
Source: Surg Endosc. 2018 Jul 25;32(10):4078–86. doi: 10.1007/s00464-018-6092-5 (PMC6132885; doi:10.1007/s00464-018-6092-5)
Supplement: Supplementary file 1 — Supplementary material 1 (DOCX 14 KB) [file 464_2018_6092_MOESM1_ESM.docx]

**Supplementary File**

ICD-10 codes Cholecystitis:

K80.0: Calculus of gallbladder with acute cholecystitis

K80.1 Calculus of gallbladder with other cholecystitis

K80.4 Calculus of bile duct with cholecystitis

K81.0: Acute cholecystitis

K81.8: Other cholecystitis

K81.9: Cholecystitis, unspecified

Admission code:

11 – 13: Elective

21 – 28: Emergency

OPCS-4 Procedure codes:

*Treatment cholecystitis:*

J18.1: Total cholecystectomy and excision of surrounding tissue.

J18.2: Total cholecystectomy and exploration of common bile duct.

J18.3: Total cholecystectomy NEC.

J18.4: Partial cholecystectomy and exploration of common bile duct.

J.18.5: Partial cholecystectomy NEC.

J18.8: Other specified excision of gallbladder.

Y50.8: Other specified approach to abdominal cavity

Y75.1: Laparoscopically assisted approach to abdominal cavity.

Y75.2: Laparoscopic approach to abdominal cavity NEC.

Y75.8: Other specified minimal access to abdominal cavity.

Y75.9: Unspecified minimal access to abdominal cavity.

J37.2: Operative cholangiography through cystic duct.

J37.3: Direct puncture cholangiography.

J37.4: Operative choledochoscopy

*Cholecystostomy*

J21.2: Drainage of gallbladder

J24.1: Percutaneous drainage of gallbladder.

*ERCP*

J38.1-J38.2: Endoscopic sphincterotomy of sphincter of Oddi and removal of calculus HFQ/insertion of tubal prosthesis in bile duct.

J38.8: Other specified incision of sphincter of Oddi

J38.9: Unspecified endoscopic incision of sphincter of Oddi.

J40.2: Endoscopic retrograde insertion of tubal prosthesis into bile duct NEC

J40.8: Other specified endoscopic retrograde placement of prosthesis in bile duct

J40.9: Unspecified endoscopic retrograde placement of prosthesis in bile duct

J41.1: Endoscopic retrograde extraction of calculus from bile duct

J41.2: Endoscopic dilation of bile duct NEC

J41.3: Endoscopic retrograde lithotripsy of calculus of bile duct

J41.4: Endoscopic retrograde photodynamic laser therapy of lesion of bile duct

J41.8: Other specified other therapeutic endoscopic retrograde operations on bile duct

J41.9: Unspecified other therapeutic endoscopic retrograde operations on bile duct

*Bile Duct Reconstruction*

J27.2 – J27.4: Partial excision of bile duct and anastomosis of bile duct to duodenum/jejunum/ end to end anastomosis of bile duct.

J29.1: Anastomosis of hepatic duct to transposed jejunum and insertion of tubal prosthethis HFQ.

J29.2: Anastomosis of hepatic duct to jejunum NEC.

J30.1 – J30.3: Anastomosis of common bile duct to duodenum/transposed jejunum/jejunum NEC.

J32.1 – J32.9: Reconstruction of bile duct/re-anastomosis of bile duct/other specified repair of bile duct/unspecified repair of bile duct.

J37.8: Other specified other open operations on bile duct.

J37.9: Unspecified other open operations on bile duct.
